# Digoxin in amyloidosis: Is it associated with a greater incidence of arrhythmogenic potential?

**DOI:** 10.1002/joa3.12775

**Published:** 2022-09-05

**Authors:** Ramzi Ibrahim, Chelsea Takamatsu

**Affiliations:** ^1^ Department of Medicine Banner University Medical Center‐University of Arizona Tucson Arizona USA

Letter to the Editor:

Digoxin is notoriously perceived to be associated with a greater arrhythmogenic potential in patients with amyloidosis, a misconception mainly rooted in a historical case report in 1961 and an in‐vitro study in 1981.^1^, ^2^ It has since then been discouraged due to a presumed increased risk of toxicity and sensitivity. However, given the scarcity of current supporting data, we believed this warranted a contemporary review of the literature. This review assesses the incidence of digoxin‐associated arrhythmogenicity in patients with amyloidosis by synthesizing cumulative quantitative data on the total frequency of associated arrhythmias. A literature review using the terms ((amyloidosis [mesh] OR amyloid* [tiab]) AND (digoxin [mesh] OR digitalis [mesh] OR digitalis glycosides [mesh] OR digoxin [tiab] OR digitalis [tiab])) yielded 29 488 studies total from multiple databases. Studies were included only if they were clinical trials or observational studies which reported digoxin use in the setting of patients with known amyloidosis. All other types of studies were excluded from this analysis. Studies were independently evaluated by two independent physicians. Only two observational studies were included given that the rest of the studies did not meet the inclusion criteria.^3^, ^4^ Continuous values were described as absolute values and respective percentages. Within these 2 observational studies, a total of 202 patients with known amyloidosis were identified. 165 patients had known AL amyloidosis, and 37 had ATTR (13 hereditary and 24 wildtype). A mean duration of 5.5 months of digoxin use was observed with a mean GFR estimated at 57.65. 165 patients (81.7%) were on digoxin for atrial fibrillation/flutter and 37 (18.3%) for heart failure. Pooled number of arrhythmias resulted in sinus bradycardia in 5 patients (2.5%), asystole in 5 patients (2.5%), PEA in 4 patients (2%), junctional rhythm in 7 patients (3.5%), ventricular tachycardia in 7 patients (3.5%), ventricular fibrillation in 2 patients (1.0%), 3rd degree AV block in 1 patient (1.0%), breakthrough atrial fibrillation without RVR in 1 patient (1.0%), and non‐sustained ventricular tachycardia in 1 patient (1.0%). Given the scarcity of available data on this topic, this contemporary review identified 2 observational studies that explored the arrhythmogenic potential of digoxin in patients with known amyloidosis. Based on our findings, up to 4% of patients with amyloidosis will develop an arrhythmia from digoxin, which is consistent and not significantly greater than what is known through current literature regarding digoxin‐induced arrhythmias in patients without known amyloidosis.^5^ This study delineates a gap in available data in the literature for supporting the long‐perpetuated theory that digoxin use is associated with higher arrhythmogenic potential in patients with amyloidosis.

## CONFLICT OF INTEREST

None. The author has no conflict of interest. This research did not receive any specific grant from funding agencies in the public, commercial, or not‐for‐profit sectors. No disclosures.
